# Why Do Women Pretend to Be Men? Female Gender Swapping in Online Games

**DOI:** 10.3389/fpsyg.2022.810954

**Published:** 2022-05-12

**Authors:** Liling Zhou, Ning Han, Zeran Xu, Corlyn Brian, Siraj Hussain

**Affiliations:** ^1^School of Journalism and Communication, Wuhan University, Wuhan, China; ^2^School of Foreign Languages, Changchun Guanghua University, Changchun, China; ^3^Annenberg School for Communication and Journalism, University of Southern California, Los Angeles, CA, United States; ^4^Department of Sociology, Bahauddin Zakariya University, Multan, Pakistan

**Keywords:** gender, online games, gender swapping, gender role, avatar, *Tanbi*

## Abstract

This research explored the influencing factors of gender swapping among female players in online games and their impact on online gaming behavior. Based on an online survey of 3,658 female players in China, we found that perceived benefits and the *Tanbi* tendency, a psychological indulgence in enjoying novels, comics, or series on love and sex between attractive males, were the most important factors for female players to employ male avatars. Sexual orientation, perceived anonymity, and perceived tolerance also had a significant influence on gender swapping. Different from the practical benefits perceived by men who use female avatars in online games, the perceived benefit for female players who use male avatars was to avoid gender discrimination. In order to obtain more freedom and fairer treatment, they chose male avatars for a better experience. Female players with a higher degree of gender swapping showed a stronger aggressiveness and dominant “hyper-masculinity” behavior tendency in the game. Though online virtual worlds may be a convenient place for females to experience gender equality through gender swapping, the findings of this study suggest that gender swapping in games may, to some extent, perpetuate or even reinforce gender stereotypes in the real world.

## Introduction

The development of the Internet has made it easier for people, regardless of their gender, to freely interact with others through online games. This has led to a growing number of online gamers worldwide. The COVID-19 pandemic has further increased the importance of virtual worlds. The demand for video games has increased, and many people have turned to online games to cope with stress and forget about the pandemic (Şener et al., 2021). Currently, there are an estimated 1 billion internet gamers worldwide (Clement, 2021). Female players are becoming increasingly prevalent in online gaming environments around the world, and many females are spending more time on gaming (Google and Niko Partners, 2020). In China, the market revenue of online games has exceeded 41 billion dollars. And there has also been an upsurge in the number of female gamers online. Around 390 million female gamers were reported in China as of February 2020 which accounts for 45.6% of the overall gamers in China (Thomala, 2021). However, players who reveal themselves as females in online games are known to be vulnerable as they are often mistreated by other players (male players) (Cote, 2017; McLean and Griffiths, 2019; Tang et al., 2019; Hao et al., 2020). These experiences may lead to female gamers’ choosing male avatars in online games, a practice known as gender swapping.

Gender swapping in online games refers to the behavior of game players using avatars of different genders (Tseng et al., 2018). This phenomenon is commonly seen in massively multiplayer online role-playing games (MMORPGs or MMOGs for short). Due to the nature of MMOGs, gamers can play various virtual roles through virtual gender swapping. However, gamers were mainly male in early stages of gaming history (McClure and Mears, 1984; Krotoski, 2004). This led to the fact that most studies on virtual gender swapping focus on male players (Hussain and Griffiths, 2008; Huh and Williams, 2010; Lehdonvirta et al., 2012; Lou et al., 2013; Song and Jung, 2015). Therefore, there is still a research gap regarding the reasons why female players, particularly female players in China, disguise themselves with male avatars in online games, as well as the consequences of this behavior in real life.

The question is, why do females use male avatars in online games? What does this gender-swapping behavior mean? What impacts does gender swapping in online games have on gamers in real life? Based on these questions, this research conducted an empirical study on female players, focusing on the motivations and results of their gender swapping.

## Literature Review and Hypotheses

Gender swapping can be easily achieved in the virtual worlds of gaming. The findings from an online survey conducted in the United States reveal that 54% of male players and 68% of female players swapped their gender in online games (Hussain and Griffiths, 2008). As for the reasons for changing genders in games, existing research has provided the following explanations: to express the real self (Huh and Williams, 2010), to express the other self or the “second self” (Kafai et al., 2010), for fun (Hussain and Griffiths, 2008), and to obtain practical benefits (Bartle, 1996; Song and Jung, 2015). Some scholars have studied gender swapping from an impression management perspective (DeAndrea et al., 2012), while others have regarded it as the behavior of sexual minorities in virtual spaces (Huh and Williams, 2010).

However, as mentioned earlier, the existing gender swapping related studies have mainly focused on the gender change behaviors of male gamers, but the reasons for gender swapping among males do not necessarily apply to women. For example, Song and Jung (2015) argued that female players might have different motivations and behave differently after gender swapping. According to Huh and Williams (2010), females are more likely than males to change gender online as a way of in-game identity exploration to challenge traditional gender norms and stereotypes of females in the real world.

### Avatar and Gender Role

According to Goffman, the body serves as an intermediary between self-identity and social identity. In virtual worlds, “our social identities tend to be governed by a general desire to present ourselves as “normal” people worthy of playing a full part in society” (Shilling, 1993, p. 86). In online games, bodies are expressed through customized avatars. An avatar is “the virtual embodiment of users in online games” (Banakou and Chorianopoulos, 2010, p. 4). However, an avatar is not just a “costume” in many cases, but considered as an “entire self-representation” (Yee and Bailenson, 2007) or a “second self” (Kafai et al., 2010) of the player. Players create avatars to reflect their offline identities, and each avatar has the player’s own personality (Kafai et al., 2010).

Gender roles refer to “behaviors, expectations, and role sets defined by society as masculine or feminine which are embodied in the behaviors of the individual man or woman and culturally regarded as appropriate to males or females” (O’Neil, 1981, p. 203). In other words, gender roles are stereotypes that require a person to act in a certain way based on society’s expectations related to gender. The varying degrees of power that men and women hold in society can be connected to gender roles. For instance, men are expected to experience greater power (masculine or dominant roles) than women in society (Blackstone, 2003, p. 337; McKeen, 2005).

Researchers have shown that traditional gender-role oriented individuals were more likely to avoid cross-gender behavior, even at monetary costs (Bem and Lenney, 1976). Gender-role orientation was defined as “the extent to which individuals’ self-perceptions conform to the culture’s definitions of maleness and femaleness” (Bem, 1981, p. 355; Lee, 2007, p. 518).

Based on the above studies, it is reasonable to assume that a female player is more likely to choose a male avatar in online games if she perceives herself as having those traits that are considered masculine by mainstream social norms, such as forcefulness, dominance, risk-taking, and leadership abilities.

Hypothesis 1: Females with less traditional gender-role orientations are more likely to engage in gender swapping in online games.

### Sexual Orientation and *Tanbi* Tendency

Previous studies have shown that the sexual orientation of certain players plays a crucial role in their gender-swapping behavior in online games. For example, a survey conducted on 6,122 players in EverQuest II (a MMOG designed by Sony) reveals that virtual gender swapping is more common among players who report same-sex attraction (Huh and Williams, 2010). However, some scholars argue that this behavior arises from a desire to appreciate the physical attractiveness of opposite sex characters (Hussain and Griffiths, 2008; Fahs and Gohr, 2012).

In this study, the appreciation of the beauty of the opposite sex refers to females’ aesthetic perception of males since we take female gender swappers as research subjects. In this regard, we decided to use the term “*Tanbi*” instead of the term “aesthetics” because “*Tanbi*” is suitable to cover both situations. *Tanbi* refers to a psychological indulgence in enjoying novels, comics, or series on love and sex between attractive males, which is not only associated with same-sex tendencies, but also with the strong aesthetic demand for the physical beauty of males (Wang, 2011). Combined with previous research, *Tanbi* tendency is likely to be another important factor leading to gender swapping.

The word “*Tanbi*” originates from the Japanese word “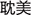,” which originally meant aesthetic or romantic indulgence in the beauty of art. Later, it became a term that refers to indulging in the beauty of love between handsome males without involving reproduction. In the 1960s, novels, especially comics, depicting love between attractive males, appeared in Japan, with large groups of young female readers, who called themselves *Fujoshi* (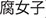), a term used to describe women who have a special hobby of reading comics or novels about romantic love stories between attractive men. In the late 1980s, *Tanbi* comics spread to China, which led to the further development of *Tanbi* culture in China. *Tanbi* novels created by Chinese writers appeared online in the 1990s (Wang, 2011), and literature of this genre in China also focuses on romantic relationships between men. *Tanbi* literature has always been written by women, and there is a sizable audience for it. However, this does not necessarily mean that the authors or the readers of this genre are sexually attracted to people of the same sex. Women who write or are passionate about *Tanbi* are known as “*Fujoshi*.”

A few Chinese scholars have studied and elaborated on the development of *Tanbi* in China and its influence on women. Some scholars conclude that women who are passionate about *Tanbi* are sexually attracted to people of the same sex (Ruan, 2008). Other researchers, however, believe that it has nothing to do with sex orientation. Instead, they believe that these women are expressing their inner wishes and expectations through the male “body,” implying that the passion for *Tanbi* among women is motivated by a desire for purer love, secret sexual urges, or psychological needs such as curiosity. The fantasy of *Tanbi* is a conduit for women to express their wants through depictions of same-sex feelings or sexual expressions, as well as a desire for a society with greater equality and freedom (Wang and Liu, 2008). Therefore, it may be seen as a resistance to the traditional patriarchal society and heterosexual power. In *Tanbi* related works, for example, same-sex love is no longer treated as perverse, but as normal as heterosexuality. The openness, interactivity, and anonymity of the modern internet have also aided in the proliferation of *Tanbi* culture in China.

Despite the proliferation of *Tanbi* culture in China, *Tanbi* and same-sex related topics are still taboo in public. People who are unable to reveal their secret hobbies in real life can interact socially through the internet, seek pleasant interactions, and find their “utopia” in virtual worlds. The anonymity of the online world provides them with a more secure and welcoming environment, devoid of discrimination, external pressure, or condemnation for not fitting social moral standards. The MMOG world has provided a convenient place for *Fujoshis* to construct and experience their own *Tanbi* game roles. Not only can they portray their feelings for the same sex, but they can also play the male roles themselves, interact closely with other male players, and seek a welcoming environment to air their views freely.

In sum, sexual minorities, such as females with lesbian or *Tanbi* tendencies, can easily make use of gender swapping to avoid discrimination and external pressure associated with their sexual orientation or preferences, to appreciate gazing at men rather than being gazed at, and even to create more ideal male images according to their own aesthetic standards and construct the relationships between men in the online game world. In contrast, sexual majorities are less likely to have these motives. Therefore, the following hypotheses can be reasonably put forward:

Hypothesis 2: Female players with lesbian tendencies have a higher degree of gender swapping behavior than female players with heterosexual orientation.

Hypothesis 3: Female players with heterosexual orientation have a lower degree of gender swapping behavior than female players with lesbian tendencies.

Hypothesis 4: Female players with *Tanbi* tendencies have a higher degree of gender swapping behavior.

### Perceived Benefits

Men employ female avatars for specific benefits, according to current studies on gender swapping in online games. Male players in virtual worlds are motivated by the desire to gain more practical benefits, such as more money or weapons, to be treated more politely, or to avoid being targeted and attacked by other players (Hussain and Griffiths, 2008; Song and Jung, 2015). In male-dominated online gaming environment, although female identities have many disadvantages, such as being perceived as incompetent for competitive tasks (Kaye et al., 2017; Perry, 2021), their identities also have their positive social advantages. An example of such advantage is that male players tend to be friendlier to female players.

While existing research focuses on male players, whether female players swap gender for similar motives remains a research gap. According to an online survey conducted on 293 respondents from 30 countries, women in online games face both general and sexual harassment. Some women may withdraw from games due to such treatments. This leads to the adoption of masculine screen names and avatars to avoid being abused or harassed by male players (Fox and Tang, 2016). Males are more likely than females to engage in harassment in online video games. And female avatars are the very targets of sexual harassment in most cases. To avoid being harassed, female players may swap their gender and play in disguise (Tang et al., 2019).

In most online games, female characters are often designed as sexy beauties, often scantily dressed, indicating that they are only treated as “vases” rather than competitive players. For female players, the gender label is more visible than their gaming skills or competence, which are often underestimated just because of their gender. And the females may even be subjected to gender humiliation or harassment by other players due to their gaming mistakes (Huh and Williams, 2010; Fox and Tang, 2016).

Previous studies reveal that gender inequality has been embedded in online games in multiple ways. Male dominance in video games is mirrored in game design and production, as well as the definition and classification of gamers (Cote, 2018; Vilasís-Pamos and Pires, 2021). Cote (2018) noted that video games are still a “masculinized technology.” Females, on the other hand, are often “hyper-sexualized and relegated to disempowering roles” (Perry, 2021, p. 1).

In order to overcome gender restrictions on women similar to those in real life, or to prevent male players from belittling, attacking, or harassing them, female players may swap their gender for more equal treatment and a better gaming experience (Hussain and Griffiths, 2008; Huh and Williams, 2010). In other words, a female player may perceive certain benefits of playing as a male character, although these benefits may not be material or practical, but other types of rewards. Thus, the more a female player perceives the benefits of gender swapping, the more likely she is to use a male avatar.

Hypothesis 5: Perceived benefits positively predict gender swapping for female players.

### Perceived Environmental Safety

Players’ perceptions of social tolerance for gender swapping and the security of anonymity are particularly important in relation to the adoption of gender swapping as a behavior that may cause negative treatments. The first is the perceived social tolerance for gender swapping in online games. Players may experience embarrassment or difficulty as a result of gender swapping. Male players who use female avatars, for example, may face discrimination from other players, particularly if they conceal their true gender and attempt to engage in online romantic relationships in order to be cared for and helped by other male players. Gender swapping may be considered as a form of deceit. Swapping genders might be deemed gay or mentally gender dislocated. Gender swappers in the virtual world, like sexual minorities in the real world, are frequently the target of criticism. Failure to follow gender roles can result in poor impressions and assessments from others, as well as criticism and punishment. Therefore, the acceptance or tolerance of gender swapping by the external environment could be an opinion climate valued by gender swappers.

The second is the perceived security of anonymity in online gaming environments. Song and Jung (2015) pointed out that anonymity is a prerequisite for gender swapping. Compared to offline reality, virtual worlds may provide a more anonymous and secure environment. Although from a certain aspect, the lack of identity clues may make social relationships in virtual worlds seem empty and fragile, the anonymity of the internet allows them to safely shed their real-world identities and experience alternative identities in the virtual world.

Hypothesis 6: The higher the perceived tolerance for gender swapping, the higher the degree of gender swapping.

Hypothesis 7: The higher the perceived anonymity of the environment, the higher the degree of gender swapping.

### Proteus Effect and Hyper-Masculinity

Bartle (1996) implied gender differences in the way gamers play. He argued that men are more likely to be achievers and women are more likely to be socializers. Yee (2006) further confirmed that female gamers play games primarily for social purposes and are keen to make new friends. The question is whether the player’s conduct will alter as a result of changing gender.

Deindividuation theory states that when individuals are in an anonymous environment that lacks personal information such as identity clues, they may behave differently than usual. Yee and Bailenson (2007) found that regardless of their actual image, players who adopted tall, handsome male avatars appeared more confident and intimate in their interactions with other players in online games. They also appeared more confident and powerful when it came to negotiating tasks, just as a tall, attractive man would do in the real world. Yee and Bailenson (2007) defined this pattern of behavior as the Proteus Effect, meaning that individuals tend to act according to their digital self-representation, regardless of how others perceive them. According to this proposition, when gamers use different avatars, they tend to act in ways that match the personality traits of their avatars.

Yee and Bailenson (2007) also pointed out that there may also be a gender-based Proteus Effect in online games. Men, for example, will act in ways that are more in line with feminine norms when they utilize female avatars. Subsequent studies have provided some evidence to support this claim. According to Huh and Williams (2010), female gender swappers exhibit more masculine behavior patterns, even more so than male players. For example, female gender swappers take part in more PvP combat and less text chatting than female non-swappers. Whereas, female non-swappers chat more than any other group, female gender swappers chat the least of any group, including the men, suggesting that the female swappers are engaging in hyper-masculine behaviors to act their role as males (Huh and Williams, 2010). Even though male gender swappers keep their own masculine features in terms of combat behavior patterns, such as jumping, they replicate conventional feminine gestures and imitate their assumed female speech style, according to a study on gender swapping of male gamers (Martey et al., 2014).

Lehdonvirta et al. (2012) argued that traditional gender role descriptions as perceived by players shape their expectations of individuals and thus influence their behavior. When playing a new gender role, gender swappers may speculate on the behavioral characteristics of the other gender and often follow social stereotypes about being male or female, so they tend to overstate gender differences and reinforce gender stereotypes. In this sense, virtual worlds may become a place where “hyper-masculinity” and “hyper-femininity” grow. Hyper-masculinity refers to exaggerated “masculinity,” specifically the desire for aggression, violence, and dominance (Scharrer, 2004). Based on the Proteus Effect hypothesis and existing research on the consequences of gender swapping in online games, this paper further proposes the following hypothesis:

Hypothesis 8: The higher the degree of gender swapping behavior, the stronger the hyper-masculinity of female players in the game.

## Materials and Methods

### Participants and Procedure

The purpose of this study is to explore the gender swapping behaviors of female players, so a popular massively multiplayer online game (MMOG) known as “Final Fantasy XIV” (referred to as “FF14” for short) was chosen as the research site. Square Enix, a Japanese game company, released the game in 2010 and launched it in China in 2014. According to the official website of FF14, the game has over 25 million players worldwide and a 9.5/10 IGN rating. The Chinese server 5.57 version of FF14 has eight races, including: Hyur, Elezen, Lalafell, Miqo’te, Roegadyn, Au Ra, Viera, and Hrothgar. Each race, with completely different appearances, has its own unique characteristics, such as attractive Lalafell, towering Elezen, fierce Au Ra, mysterious Miqo’te, etc. Players can freely experience up to 29 different fates in this game. FF14 offers a variety of communication channels for social engagement, including private chat, creating teams, chatting, yelling, and shouting.

Because of its cinematic storyline, non-pornographic character setup style, and a wide range of societal themes, the game has attracted a wide range of female users in recent years. There was an even split between male and female players of the game as of 2017 (Gibson, 2017). The gaming character in FF14 is known as the Warrior of Light. The Warrior of Light is frequently depicted as a man in official settings and promotional materials. Players may also use the “Fantasia vial” (a potion that grants users the opportunity to change the appearance or gender of the avatar in FF14). Since the price of a Fantasia vial is relatively low, and there are multiple channels for obtaining it, gender swapping is easy to achieve, and it is also relatively common in FF14.

After conducting a small pilot study using the in-depth interview method, an online survey of FF14 users was conducted in China in April 2019. The questionnaire was publicized and distributed through the FF14-related Weibo accounts “Aiziya Joke Station” and “Talk to Haidelin” operated by the players themselves. At the same time, a small number of lottery tickets were given as rewards. The questionnaire was reposted 1,703 times spontaneously, with 133 comments. A total of 4,377 valid samples were gathered using a voluntary convenience sample that permitted players to repost for snowball sampling, all of whom were FF14 game users. The total sample included 719 male users and 3,658 female users. This study uses the 3,658 female respondents obtained as the research subjects.

Our samples are all from China. Among the 3,658 female respondents, 75.5% were under the age of 24, 60.0% were students. 42.2% claimed themselves as heterosexual, 6.5% as gay, 37.3% as bisexual. Out of all the valid samples, 2,860 reported gender swapping behaviors in FF14, accounting for 78.0% of the overall data, indicating that gender swapping is a common behavior in FF14.

### Measures

#### Gender Swapping

In this study, gender swapping was operationally defined as the length and frequency of use of male avatars by female gamers. It consisted of two items on a 5-point scale: “I spend more time using male avatars in online games,” and “I use male avatars more often than female avatars in online games.” The Pearson’s correlation coefficient was 0.883 (*M* = 3.53, *SD* = 1.291), indicating good internal consistency.

#### Gender-Role Orientation

The Bem Sex Role Inventory (BSRI) has been widely used to measure gender roles. BSRI includes three measurement parts: masculine items, feminine items, and neutral items (Lee, 2007). Since this study only examined the effect of female players’ masculinity, a typical manifestation of less gender-role orientation (non-gender-typed), on their gender swapping behavior, only 20 items in the BSRI about masculinity were selected. Respondents were asked to rate the extent to which a set of masculinity-related adjectives or phrases applied to them, using items on a 5-point scale. Using principal component factor analysis, three factors with good reliability and validity were extracted from the 15 items: autonomy, leadership, and aggressiveness. Autonomy included “self-reliant,” “willing to take a stand,” “independent,” “self-sufficient,” “individualistic,” “makes decisions easily,” and “assertive,” with a Cronbach’s alpha of 0.872 (*M* = 3.63, *SD* = 0.815). Leadership included “acts as a leader,” “leadership ability,” “dominant,” “willing to take risks,” and “competitive,” with a Cronbach’s alpha of 0.839 (*M* = 2.44, *SD* = 0.842). Aggressiveness included “aggressive,” “forceful,” and “ambitious,” with a Cronbach’s alpha of 0.794 (*M* = 2.85, *SD* = 0.930).

#### Sexual Orientation and *Tanbi* Tendency

Sexual orientation was a nominal variable. Respondents chose from the following five options: heterosexual (1,543, 42.2%), homosexual (237, 6.5%), bisexual (1,366, 37.3%), asexual (273, 7.5%), and other (239, 6.5%). The *Tanbi* tendency was measured by four 5-point scale items: “I enjoy the appearance and body of male avatars,” “I like stories associated with *Tanbi* themes,” “I want to write or have written blogs or novels with *Tanbi* themes,” and “I often fantasize about developing a romantic relationship with other men (real or imaginary).” The Cronbach’s alpha was 0.734 (*M* = 3.67, *SD* = 0.845).

#### Perceived Benefits

Perceived benefits for female gender swappers were measured by two items: “I think the use of male avatars is less restrictive and freer than using female avatars,” and “When I use a male avatar, my gaming experience is better than using a female avatar.” The Cronbach’s alpha was 0.777 (*M* = 2.68, *SD* = 1.061).

#### Perceived Environmental Safety

Perceived environmental safety measured the perception of anonymity and other players’ tolerance of gender swapping in online games, indicating gamers’ perceptions of the safety of the external environment for gender swapping. The items measured were modified from two items by Song and Jung (2015): “I think my actions or decisions are concealed in online environments (*M* = 3.47, *SD* = 1.004),” and “I think other players in the game don’t care about my real physiological gender (*M* = 3.88, *SD* = 0.898).” The corresponding variables were “perceived anonymity” and “perceived tolerance.”

#### Hyper-Masculine Gaming Behaviors

Six items were used to measure hyper-masculine gaming behaviors, and three main components were generated, namely “aggressiveness,” “dominance,” and “competitiveness,” representing three types of hyper-masculine gaming behaviors. “Aggressiveness” contained three 5-point scale items: “Compared with chatting or hanging up in the games, I prefer to play game instances or other combats,” “I always go through levels to play the current difficult level,” and “I like to study game strategies to improve my operation and output.” The Cronbach’s alpha was 0.719 (*M* = 3.22, *SD* = 0.941). “Dominance” contained two 5-point scale items: “Through my own efforts, I achieved what most players did not achieve,” and “Most of the time, I am the commander of team activities,” indicating a player’s benchmark achievement and dominant characteristics. The Pearson’s correlation coefficient was 0.658 (*M* = 2.30, *SD* = 0.893). “Competitiveness” included one item: “I love to beat other players in PVP and enjoy winning (*M* = 2.69, *SD* = 1.19).”

## Results

### The Influencing Factors of Female Players’ Gender Swap

To explore the reasons for gender swapping among women, a hierarchical multivariate regression analysis was conducted using gender-role orientation, sexual orientation and *Tanbi* tendency, perceived benefits and safety as three sets of independent variables. The results showed that all three sets of independent variables had significant predictive power for gender swapping and could explain the overall variation in the dependent variable by 30.3%. The collinearity diagnosis results were acceptable with values of tolerance ranging from 0.501 to 0.898. The detailed results are presented in Table 1.

**TABLE 1:** Multivariate regression analysis for predicting gender swapping.

Independent variables	Dependent variable: gender swapping					
	Unstandardized coefficients		Standardized coefficients	*t*-value (sig.)	Collinearity statistics	
				
	B	Std. error	Beta		Tolerance	VIF
(Constant)	0.131	0.180		0.732		
**Gender-role orientation**						
autonomy	0.128	0.033	0.079	3.853***	0.676	1.479
leadership	–0.016	0.036	–0.010	–0.437	0.520	1.923
aggressiveness	0.001	0.034	0.001	0.038	0.501	1.995
*R*^2^(%)	1.8***					
**Sexual orientation and *Tanbi* tendency**						
SO1 (1 = heterosexual)	–0.118	0.047	–0.045	−2.542*	0.898	1.114
SO2 (1 = same-sex)	0.347	0.086	0.073	4.045***	0.891	1.122
*Tanbi* tendency	0.389	0.028	0.253	13.962***	0.873	1.145
Increased *R*^2^(%)	14.0***					
**Perceived benefits and safety**						
Perceived benefits	0.468	0.022	0.381	21.113***	0.884	1.132
Perceived anonymity	0.063	0.024	0.049	2.641**	0.847	1.180
Perceived tolerance	0.113	0.026	0.078	4.281***	0.863	1.159
Increased *R*^2^(%)	14.8***					
***R*^2^(%) total**	30.6***					
**Adj. *R*^2^(%) total**	30.3***					

N = 2,428; *p <0.05, **p < 0.01, ***p <0.001.

#### The Impact of Gender Role on Gender Swapping of Female Players

Among the three dimensions of masculinity, only “autonomy” had a statistically significant effect on gender swapping (β = 0.079, *p* < 0.001). However, “leadership” and “aggressiveness” had no significant effect on gender swapping. Hypothesis 1 was partially confirmed. In general, the explanatory power of gender-role orientation on gender swapping behavior was only 1.8%, indicating that from a practical perspective, gender-role orientation had little influence on women’s gender swapping.

#### The Influence of Sexual Orientation and *Tanbi* Tendency on Gender Swapping

The results confirmed the negative effect of heterosexual orientation on gender swapping (β = −0.045, *p* < 0.05), the positive effect of same-sex orientation on gender swapping (β = 0.073, *p* < 0.001), and the positive effect of *Tanbi* tendency on gender swapping (β = 0.253, *p* < 0.001). Hypotheses 2, 3, and 4 were confirmed. Sexual orientation and *Tanbi* tendency had relatively strong explanatory power for gender swapping, which accounted for 14% of the total variation of the dependent variable. Among them, the influence of *Tanbi* tendency was particularly important, and its standardized regression coefficient reached 0.253.

#### The Impact of Perceived Benefits and Safety on Female Gender Swapping

Multivariate regression analysis results revealed that perceived benefits and safety were the strongest predictive independent variables set for gender swapping and could explain 14.8% of the total variation in gender swapping. Among them, perceived benefits had the strongest explanatory power for gender swapping (β = 0.381, *p* < 0.001), and the effects of both perceived gender swapping tolerance and perceived environmental anonymity were statistically significant, with standardized regression coefficients of β = 0.078 (*p* < 0.001) and β = 0.049 (*p* < 0.01), respectively. Hypotheses 5, 6, and 7 were confirmed.

### Gender Swapping and “Hyper-Masculinity” of Female Players

Existing studies suggest that gender swapping might cause players to adopt more exaggerated and stereotyped behaviors (Hussain and Griffiths, 2008; Song and Jung, 2015). This paper examined the relationship between gender swapping and “hyper-masculine” game behaviors of female players (see Table 2). Pearson product-moment correlation analysis showed that gender swapping was not statistically correlated with competitive behavior, but was positively correlated with aggressive and dominant behavior. Hypothesis 8 was partially confirmed.

**TABLE 2:** Correlation analysis summary of gender swapping and three types of hyper-masculine behaviors.

Variables	Gender swapping	Aggressive	Dominant	Competitive
Gender swapping	1 (2,860)	0.043* (2,860)	0.039* (2,860)	−0.011 (2,860)
Aggressive	0.043* (2,860)	1 (3,658)	0.562*** (3,658)	0.298*** (3,658)
Dominant	0.039* (2,860)	0.562*** (3,658)	1 (3,658)	0.267*** (3,658)
Competitive	−0.011 (2,860)	0.298*** (3,658)	0.267*** (3,658)	1

Sample sizes are shown in the brackets; *p < 0.05, ***p<0.001 (2-tailed).

Among the variables, the correlation coefficient between gender swapping and aggressive behavior was 0.043 (*p* < 0.05), and the correlation coefficient between gender swapping and dominant behavior was 0.039 (*p* < 0.05). According to the criterion for judging the degree of association in correlation analysis (Elliot and Woodward, 2016), the absolute values of the above two correlation coefficients were much lower than 0.40, indicating that the associations between gender swapping and aggressive and dominant behaviors were very weak. Given the large sample size of this study, the statistical significance of the correlations may not be reliable enough. Considering their weak coefficients, these correlations may not be of much practical value.

## Conclusion and Discussion

### Several Explanations for Why Women Take on Male Personas in the Game World

In the MMOG world, avatars display clues to a player’s self-identity and orientations. The results of this paper indicated that perceived benefits and *Tanbi* tendency are the most important factors for gender swapping among female players in FF14. In addition, sexual orientation, perceived environmental anonymity, perceived tolerance for gender swapping, and the autonomy dimension in masculinity, which represents a less gender-role orientation for females, also had a significant impact on the degree of female gender swapping in the game.

However, it should be noted that in this research, the operationalization of practical benefits of gender swapping refers to female players’ avoidance of and resistance to gender stereotypes that exists both online and offline. Thus, the definition of “perceived benefits” in this study differs significantly from that in prior studies, which focused on the “tricky” practical goals among male players, such as acquiring extra money or weaponry, or avoiding being attacked by other gamers. It is safe to conclude that men are more likely to change gender in games to gain practical benefits, while women are more likely to change gender for more “pure” goals, such as to break through the bondage of gender roles or stereotypes, which implies a certain degree of gender liberation.

Perceived anonymity and perceived tolerance positively predicted gender swapping, suggesting that overt gender swapping is still seen as socially risky by gender swappers, who still worry about being identified or not being accepted. The anonymity of the online world enables players to conceal their real offline identity clues, allowing players to experience male identity more freely in the virtual game space. Under the protection of online anonymity, women can experience the freedom and superiority of male identity. However, the online world is obviously not a utopian gender-free space. Behind the facades and anxiety is silent and deep-rooted discrimination.

Another notable finding was the influence of *Tanbi* and sexual orientation on female gender swapping. *Tanbi* and same-sex tendencies positively predicted gender swap, while heterosexuality negatively predicted gender swap. Previous research has confirmed the influence of aesthetic needs to appreciate the physical beauty of the opposite sex on gender swapping (Hussain and Griffiths, 2008; Fahs and Gohr, 2012), and some studies have examined the influence of same-sex orientation (Huh and Williams, 2010), but the discussion on the influence of sexual orientation is still relatively preliminary, and this factor has not been taken into account in many studies. The reason for this paper to specifically bring up the influence of *Tanbi* tendency is actually based on the findings of the in-depth interviews we conducted prior to the survey. *Tanbi* fantasy was a theme that naturally emerged in our interviews. This study examined the strong influence of *Tanbi* fantasy, which is closely, but not identically, related to physical beauty needs and same-sex orientation.

Moreover, gender role may not have as much of an impact on female gender swapping as expected. Only the impact of the autonomy dimension of masculinity has been confirmed, while leadership and aggressiveness have not. In other words, females with autonomous and independent traits are more likely to choose male avatars, while the traits around leadership or ambition have nothing to do with gender swapping. Earlier, Song and Jung (2015) found that gender schema had no significant influence on gender swapping. Combined with the finding of the influence of perceived benefits, they put forward that gamers’ motivation for gender swapping is more related to obtaining practical benefits, rather than personal identity. The current research partially supports the influence of gender roles.

### Gender Swapping and the Reproducing of Gender Inequality

The conclusions of existing studies are inconsistent on whether gender swappers have gender stereotyped behavioral patterns. Song and Jung (2015) confirmed it by revealing that male gender swappers showed greater interest in social behaviors such as chatting, socializing, and teamwork in games, which had been regarded as typical behavioral characteristics of female players. The Proteus Effect can be used as a good explanation for this phenomenon: male players reproduce stereotypes in games based on imagined female images and behaviors. Huh and Williams (2010) showed that gender swappers and non-swappers did not behave differently in games; that is, men who adopted female avatars did not act or interact in a more feminine way. However, they also found that female players disguised as males and female players with female avatars showed great differences in behavior: female players who chose male avatars were less likely to chat, fight monsters or hunt for treasure; instead they were more likely to engage in confrontational combats and high-level PVP than those who did not swap genders in the game. In other words, women who pretended to be men acted in more masculine ways.

The results of this study showed that female gamers who chose male avatars tended to display more aggressive and dominating behaviors, which were consistent with male stereotypes in the real world and the overall image of male gamers. Avatars of different genders performed different patterns of behavior in gender-typed ways: “Male” roles are more likely to be driven by the desire to achieve or to manipulate, while female roles are more likely to be driven by relationship factors. Neither competitiveness nor the joy of beating others had anything to do with the gender change of women. This proved to some extent that women do not choose male avatars to gain practical benefits such as winning the game or reaping material benefits. In other words, females who use male avatars do not use it as a game strategy to obtain game benefits.

The higher the degree of gender swapping, the more aggressive and dominant the women are, which means, the more masculine they are. This may objectively strengthen gender stereotypes, however, regardless of their subjective intentions. As Shilling (1993, p. 86) pointed out, “the way we understand, categorize, and evaluate the female and the male body is undoubtedly an important factor in legitimizing and reproducing social inequality.” How people play their roles is largely based on their imaginations and gender-typed perceptions of the opposite sex. This enables them to interact and act accordingly in virtual worlds. As a result, although female players may choose to change their gender in games to avoid the negative images and difficulties that women face in the real world, they may actually further strengthen stereotyped gender norms through their conscious imagination and deliberate imitation of men when playing and interacting with male avatars.

### Limitations and Suggestions

FF14 has more female characters due to its story and character setting. Thus, FF14 cannot stand for all types of MMOGs, and the conclusions drawn in this paper could not necessarily be applicable to other game scenarios. For example, the *Tanbi* tendency might not have much explanatory value in games like World of Warcraft. Research on gender swapping in other MMOGs is still needed in the future. In addition, a user may have multiple avatars across multiple games. Will this affect the choice of avatar gender? Again, this study is based on a volunteer sample, which may not reflect the overall female user base of MMOGs. Future studies can employ more representative samples, diverse game cases, cross game observations, and alternative data sources such as official data on players’ actual in-game behaviors and personal profiles.

Moreover, this study found a weak correlation between gender swapping and hyper-masculine behavior. Regardless of the low correlation coefficient between the two variables, it is insufficient to test the Proteus effect with correlation analysis rather than causal analysis. The Proteus effect in this case refers to whether the gender swapping of female players will lead them to behave according to the established gender stereotypes, which in turn further deepens gender stereotypes in the society. Further research into this important issue is expected to be done in the future.

Furthermore, the measurement of some variables in this study needs to be further improved. For example, both variables of perceived anonymity and perceived tolerance were measured with only one item, which could dent the validity to some extent. And the Cronbach’s alpha value of some variables was less than ideal.

Meanwhile, the proportion of bisexuals among the respondents was as high as 37.3%, and it is uncertain whether this was due to sample bias. However, studies have found that female players are three times more likely to be bisexual than males, and these bisexual players are also “hardcore” gamers because of how much time they spend on online games (Huh and Williams, 2010). Combined with the current research results on the influence of sexual orientation and *Tanbi* tendency, future research may pay more attention to the gender culture of young people.

In addition, the findings of this study are based on the data of female gamers, which does not prove that the behavior patterns described in this paper are exclusive to female gamers. We hope that more prudent comparative research can be developed to target this problem.

## Data Availability Statement

The raw data supporting the conclusions of this article will be made available by the authors, without undue reservation.

## Ethics Statement

The studies involving human participants were reviewed and approved by School of Journalism and Communication, Wuhan University, China. The patients/participants provided their written informed consent to participate in this study.

## Author Contributions

LZ, NH, and CB wrote the manuscript. CB and LZ collected the data. LZ, SH, and ZX conceived of and performed the study. LZ analyzed the data. All authors have read and agreed to the published version of the manuscript.

## Conflict of Interest

The authors declare that the research was conducted in the absence of any commercial or financial relationships that could be construed as a potential conflict of interest.

## Publisher’s Note

All claims expressed in this article are solely those of the authors and do not necessarily represent those of their affiliated organizations, or those of the publisher, the editors and the reviewers. Any product that may be evaluated in this article, or claim that may be made by its manufacturer, is not guaranteed or endorsed by the publisher.
